# Determination of Praziquantel in *Sparus aurata* L. after Administration of Medicated Animal Feed

**DOI:** 10.3390/ani10030528

**Published:** 2020-03-21

**Authors:** Elena Baralla, Maria Vittoria Varoni, Maria Nieddu, Maria Piera Demontis, Paolo Merella, Caterina Burreddu, Giovanni Garippa, Gianpiero Boatto

**Affiliations:** 1 Department of Veterinary Medicine, University of Sassari, 07100 Sassari, Italy; ebaralla@uniss.it (E.B.); varoni@uniss.it (M.V.V.); dpiera@uniss.it (M.P.D.); paolomerella@uniss.it (P.M.); caterina.burreddu@gmail.com (C.B.); garippa@uniss.it (G.G.); 2 Department of Chemistry and Pharmacy, University of Sassari, 07100 Sassari, Italy; gboatto@uniss.it

**Keywords:** praziquantel, medicated animal feed, fish muscle, residues, LC-MS/MS

## Abstract

**Simple Summary:**

The present study aimed to determine the praziquantel concentration in *Sparus aurata* muscle after oral administration of medicated feed. The in-feed treatment is commonly used in aquaculture breeding because it allows the treatment of a large fish population without stress. However, no residue limit exists for praziquantel in fish for human consumption, so the purpose of this work was to verify if this drug was able to accumulate in fish tissues after this treatment. The high-sensitivity analytical method developed in this work permitted to identify and quantify low concentrations of the drug in gilthead sea bream muscle, after the above-mentioned treatment. This method can be useful to competent authorities in evaluating the appropriate withdrawal time in fish treated with praziquantel and intended for human consumption.

**Abstract:**

Praziquantel (PZQ) is an anthelmintic drug used in humans and animals against Platyhelminthes and in aquaculture in the Far East. Medicated feed is one of the most convenient forms of oral administration of drugs in aquaculture because it allows to treat a large population of fish in an easy way. However, this treatment may lead to residues in fish intended for human consumption. In this study, a liquid chromatography with tandem mass spectrometry (LC-MS/MS) method was developed in order to verify the presence of PZQ in samples of *Sparus aurata* after oral administration of feed treated with PZQ. The method was validated according to international guidelines. It showed good recoveries, selectivity and sensitivity (LOD and LOQ were 3.0 and 9.3 ng/g, respectively), with precision and matrix effect values ≤ 15%. This method could also be applied to determine PZQ residue in other fish species and thus to evaluate the appropriate withdrawal time in treated fish intended for human consumption.

## 1. Introduction

The growth of intensive aquaculture in several countries has led to the use of different drugs in order to safeguard the wellness and health of farmed fish and prevent economic losses.

Currently, different chemotherapeutic agents are used in aquaculture, especially in Asian countries where this farming practice is particularly developed. Antiparasitic drugs are widely used in order to avoid the spread of infections in captive fish [1]. Praziquantel (PZQ) is an antiparasitic drug efficacious against Platyhelminthes; its mechanism of action is probably linked to the ability to alter membrane permeability and Ca^2+^ homeostasis through its linkage to voltage-gated Ca^2+^ channels [2]. It can be administered through different routes (oral, topical and parental) [3], but when considering intensive aquaculture, where large quantity of fish are farmed together in a facility, the most convenient administration method is the oral route through food. This method has the major disadvantage that it is not possible assume that all fish will take the same dose of drug, and the sub-therapeutic dose can eventually lead to drug-resistant infections [3]. In spite of these challenges, the in-feed administration of PZQ is currently used because of the possibility to treat a large population of captive fish without stress and in an easy way. Given the use of chemotherapeutic agents in aquaculture systems, it is very important to develop rapid, simple and sensitive analytical methods able to determine and quantify traces of these drugs in edible parts of the fish. In the literature, there are many studies describing PZQ analysis in different matrices [4,5,6]. Moreover, several studies report the quantification of PZQ in food [7,8,9,10,11,12], but no study describes the determination of PZQ in gilthead sea bream (*Sparus aurata* L.) after in-feed treatment. 

**Figure 1:**
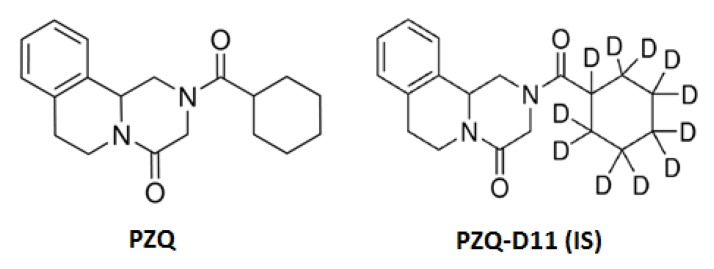
Chemical structures of praziquantel (PZQ) and internal standard (IS) PZQ-D11.

In this study, a liquid chromatography–mass spectrometry (LC-MS/MS) method was developed and validated for the determination of PZQ in fish muscle, using PZQ-D11 as internal standard (IS) (Figure 1). The validated method was used to verify the presence of PZQ and to quantify it in fish muscle after medicated feeding.

## 2. Materials and Methods

### 2.1. Materials

PZQ (2-cyclohexylcarbonyl-1,2,3,6,7-11b-hexahydro-4H-pyrazino[2,1-a]isoquinolin-4-one), PZQ-D11 (2-cyclohexyl-d11-carbonyl-1,2,3,6,7-11b-hexahydro-4H-pyrazino[2,1-a]isoquinolin-4-one) and QuEChERS tubes were purchased from Sigma Aldrich (Milano, Italy). Stock standards solutions (1 mg/mL) of PZQ and IS were prepared in methanol and stored at +4 °C. All proper dilutions were made in mobile phase when needed. All solvents used were of the highest commercial quality and were obtained from Fluka (Buchs, Switzerland). Ultrapure water was obtained using a Milli-Q water system (Millipore, Billerica, MA, USA).

### 2.2. PZQ Extraction from Fish Muscle

Five grams of *S. aurata* muscle were weighed, homogenized and transferred into a 50 mL tube. Here, 10 mL of acetonitrile containing formic acid 0.1%, 4 g of MgSO_4_ and 1 g of NaCl were added. After 10 min in ultrasonic bath to enhance the extraction of the analyte, the mixture was centrifuged at 4000 × *g* at +4 °C for 10 min.

After centrifugation, 5 mL of supernatant were transferred in a QuEChERS tube containing the dispersive phase primary secondary amine (PSA) to remove sugars and fat acids. Then, samples were vortexed for 1 minute and centrifuged at 4000 × *g* for 10 min.

Three milliliters of the supernatant were dried under nitrogen stream; the residue was reconstituted in 300 µL of mobile phase prior to being analyzed by LC-MS/MS.

### 2.3. LC-MS/MS Analysis

Analysis were performed using an HPLC ProStar300 system coupled to a Varian 310-MS triple quadrupole mass spectrometer (Varian, Palo Alto, CA, USA). The chromatographic process was carried out using a Kinetex C18 column (50 × 2.1 mm i.d., particle size 2.6 μm, Phenomenex, Torrance, CA, USA) fitted with a 2.6 μm security guard cartridge (4 × 2.1 mm i.d., Phenomenex). The mobile phase consisted of two solvents: 0.2% formic acid in water (solvent A) and acetonitrile (solvent B). A linear gradient was performed with a flow of 0.2 mL/min as follows: after 1 min at 20%, solvent B was increased to 80% in 0.06 min and maintained constant till 4 min; in the next 30 s, solvent B was decreased to 20% and kept at this percentage to re-equilibrate the system. The total run time was 8 min. Spectrometric analysis were performed using the electrospray ionization interface in positive ion mode (ESI+) under the multiple reaction monitoring mode (MRM) with the following conditions: capillary voltage, 63 V; drying gas temperature, 200 °C; nebulizer gas pressure, 50 psi; detector voltage, 1700 V. High-purity nitrogen was used as nebulizer and drying gas, while argon was used for the collision with a pressure of 2 mTorr. Collision energies (CE) were optimized to obtain the maximum detection. Two m/z transitions were used for PZQ and IS (Table 1). The retention time of PZQ was 2.5 min. 

Qualitative analysis was performed considering the selected MRM transitions and retention times criteria [13,14,15]. 

### 2.4. Method Validation

The developed method was assessed for its performance characteristics according to accepted guidelines for the validation of analytical methods [16,17]. The investigated parameters were selectivity, linearity, limit of determination (LOD), limit of quantitation (LOQ), precision, accuracy and matrix effect.

Selectivity is defined as the ability of a bioanalytical method to measure the analyte in an unambiguous way without the possibility to mix it up with other matrix components or impurities [18]. It was determined by analyzing 10 blank samples extracted as described above to verify the absence of interferences in the matrix [13]. 

Linearity was assessed through a calibration curve obtained using the extracts of spiked blank fish muscle with six different concentrations of PZQ ranging from 10 to 250 ng/g and IS (50 ng/g). Calibration curve was derived from the peak area ratios (PZQ/IS) using 1/x^2^ weighted linear least-squares regression of the area ratio versus the concentration of the corresponding standard [19].

The LOD and LOQ of the method were determined using the signal-to-noise criteria (S/N) of 3 and 10, respectively.

The precision of the method was evaluated by analyzing fish muscle spiked with PZQ (15, 100 and 200 ng/g) and IS (50 ng/g) extracted six times on the same day (intra-day repeatability) and for four consecutive days (inter-day repeatability); it was expressed as relative standard deviation (RSD), obtained as percentage of the ratio between the standard deviation and the mean value of obtained concentrations.

Accuracy was assessed as percent recovery obtained by spiking blank fish muscle samples with three different concentrations of PZQ (15, 100 and 200 ng/g) and extracting them as described above. Obtained values were compared with standard solutions of the analyte at the same concentrations that represent the 100% of recovery.

Matrix effect was determined in order to evaluate possible interferences in the matrix able to modify detector response with a decrease (ion suppression) or increase (ion enhancement) of analyte signal. For this purpose the postextraction method was used [20] and matrix effect was determined as follows:

Matrix effect % = [(RF − RS)/RS] × 100(1)

where RF is the mean peak area of reconstituted fish extract and RS is the mean peak area of the reference solution. 

### 2.5. In Vivo Application

The validated method was applied to the determination of PZQ in the muscle of *S. aurata* that received an in-feed treatment with PZQ.

Fifty specimens of *S. aurata*, ranging 50–100 g total weight, were divided into two groups (control and treated) and stocked into two different 140 L fish tanks in the Animal Facility of the Department of Veterinary Medicine. 

PZQ was mixed with standard feed for fish and given to the treated group to obtain a theoretical final PZQ dose of 50 mg/kg BW for six consecutive days. The control group was fed with the standard feed. After 30 min, the leftover feed was removed from the tanks. The treatment with medicated feed was repeated after a week. Considering the low palatability of the medicated feed, due to the bitter taste of PZQ, the real taken amount of the drug during the treatment could be lower than the theoretical one. 

The protocol treatment is shown in Table 2. The experimental protocol was approved by Italian Minister of Health and all animal experiments were performed according with the Directive 2010/63/EU (Authorization No. 526/2018-PR of 06/7/2018).

## 3. Results and Discussion

The validated LC-MS/MS method showed to be fit for the purpose of quantifying PZQ in fish matrix. Considering the complexity of the matrix, the QuEChERS extraction procedure was revealed to be fast, simple and able to give good reproducible results. The method had a good selectivity with no interfering peaks at the retention times of PZQ or IS.

A good linearity was obtained (calibration curve: y = 1.7432x + 0.0836) with determination coefficient (*r*^2^) higher than 0.99 (*r*^2^ = 0.996).The obtained LOD and LOQ were 3.0 and 9.3 ng/g, respectively, confirming the good sensitivity of the method that makes it suitable for the analysis of real samples. The described LC-MS/MS method allows to quantify lower concentrations of PZQ when compared with other HPLC-UV methods [21,22].

The analysis of the precision of the method gave intraday and interday repeatability ≤ 15%, according to the criteria of international guidelines for the validation of analytical methods [16]. The method showed very good recoveries at all the tested PZQ concentrations (Table 3).

**Figure 2:**
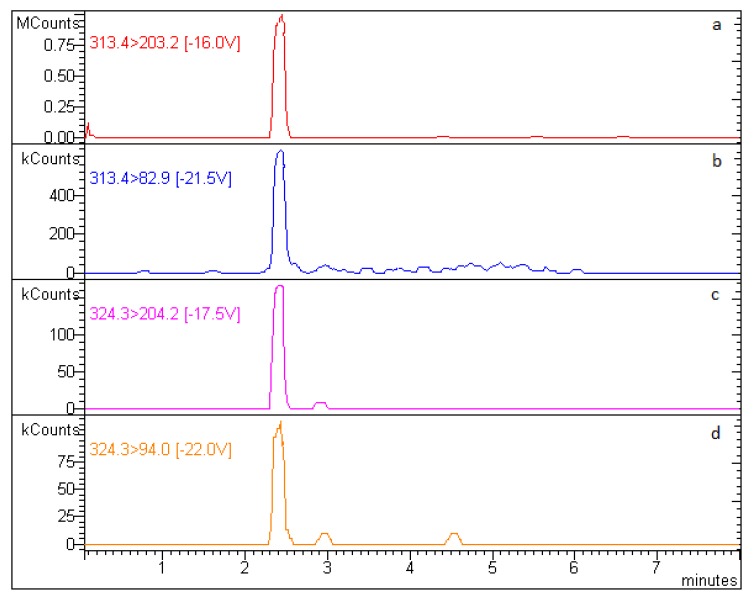
Chromatogram of a drug-free fish muscle sample spiked with PZQ (100 ng/g) and IS (50 ng/g). (**a**) main PZQ transition; (**b**) second PZQ transition; (**c**) main IS transition; (**d**) second IS transition.

Figure 2 shows the chromatogram of a PZQ-spiked fish muscle. 

Moreover, the matrix effect was within the limits provided by the guidelines (≤15%), showing the absence of matrix interference on the detector response. 

Given the results obtained for the validation parameters, the developed method was applied to determine the potential presence of PZQ in muscle of *S. aurata* after treatment with medicated feed, according to the protocol described above. 

**Figure 3:**
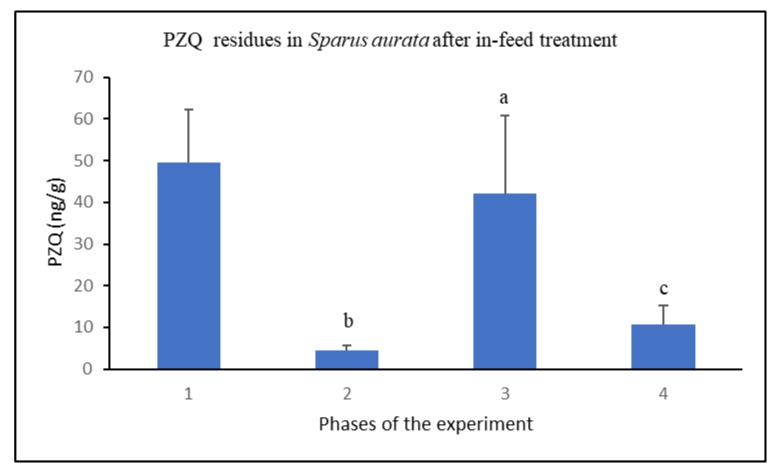
PZQ concentration in *Sparus aurata* after in-feed treatment. Data are expressed as means ± S.D. for fishes of the different phases of the experimental protocol. Letters indicate significant differences at *p* < 0.05.

**Figure 4:**
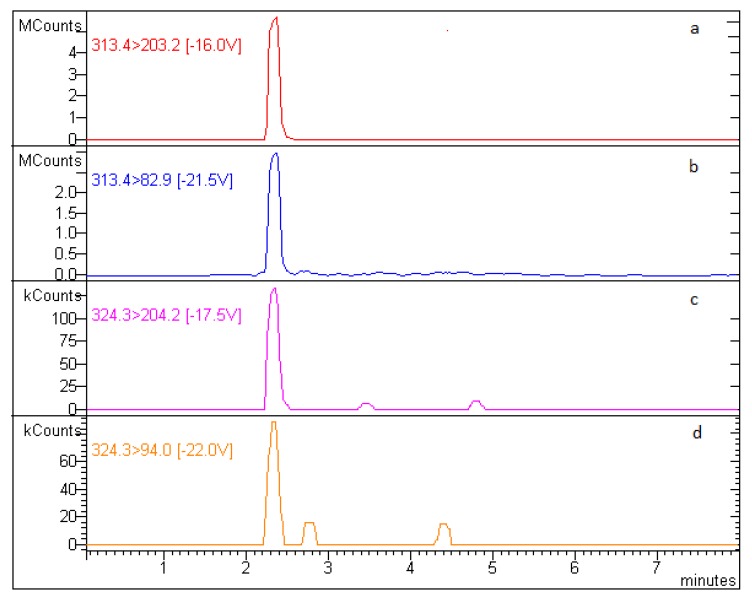
Chromatogram of *Sparus aurata* muscle sample after six days of in-feed treatment with PZQ 50 mg/kg BW. (**a**) main PZQ transition; (**b**) second PZQ transition; (**c**) main IS transition; (**d**) second IS transition.

As shown in Figure 3, PZQ residues were found in all fish samples analyzed. Figure 4 shows the chromatogram of a real sample analyzed after six days of in-feed treatment with PZQ at the theoretical dose of 50 mg/kg BW.

The mean concentration of the drug found in fish muscle after six days of treatment with PZQ was 50 ng/g, and it decreased 10-fold after a six-day suspension of the treatment. 

In the second phase of the treatment, the mean concentration of PZQ found in fish muscle was comparable to that found in the first phase of the treatment (42 ng/g). This value decreased to 10 ng/g after a second suspension of the treatment of six additional days. 

The PZQ concentrations in fish muscle were about 1000 times lower than the administered dose. These data are in accordance with those reported by Kim et al., who, after oral doses of PZQ 200 mg/kg BW for three days in rockfish, found a drug concentration of 240 ng/g in fish muscle [21].

Currently, no residue limit exists for PZQ in fish for human consumption, probably because its use is forbidden in several countries. For this reason, no detectable quantity of PZQ should be revealed in fish for human consumption [3]. Nowadays, only few studies report the analysis of PZQ residues in fish after in-feed treatment, using different doses, times of administration and fish species [21,22]. 

Most of these studies describe pharmacokinetic differences among fish species in different environmental conditions after oral treatment with PZQ doses ranging from 10 to 500 mg/kg BW [3,8].

## 4. Conclusions

Praziquantel can be used as bath or as in-feed treatment. In this work, the in-feed treatment with the antiparasitic drug was chosen as route of administration, considering that it is commonly used because it allows the treatment of a large fish population without stress.

The LC-MS/MS method developed and validated in this work is simple, fast and allows to quantify low concentrations of PZQ in fish muscle. A QuEChERS procedure was applied to extract and purify fish samples. The method was validated according to international guidelines and it was successfully applied to samples of *S. aurata* after in-feed treatment of the antiparasitic drug. 

Considering that PZQ use in fish for human consumption is forbidden in several countries, this study can be used to evaluate the absence of PZQ. On the other hand, in countries where it is normally used, this method could be applied by the competent authorities to evaluate the appropriate withdrawal time in fish treated with the PZQ and intended for human consumption.

## Figures and Tables

**Table 1:** Optimized instrumental parameters.

Analyte	Precursor Ion (m/z)	Product Ion (m/z)	Capillary (V)	Detector (V)	CE (V)
PZQ-D_11_	324.3	204.2 ^a^ 94.0	63	1700	17.5 22.0
PZQ	313.4	203.2 ^a^ 82.9	63	1700	16.0 21.5

PZQ: praziquantel; CE: collision energy; ^a^ transitions used for the quantitation.

**Table 2:** Fish treatment with PZQ (50 mg/kg).

Phases of the Experiment	Days	Experimental Protocol
0	0	Sacrifice of 5 fish
Treatment	1–6	PZQ 50 mg/kg every day
1	7	Sacrifice of 5 fish
No treatment	8–13	/
2	14	Sacrifice of 5 fish
Treatment	15–20	PZQ 50 mg/kg every day
3	21	Sacrifice of 5 fish
No treatment	22–27	/
4	28	Sacrifice of 5 fish

**Table 3:** Matrix effect, recovery and precision.

Drug	Concentration (ng/g)	Matrix Effect (%)	Recovery (%)	Repeatability (RSD %)	
				Intraday	Interday
PZQ	10	−13.7	104.6	15.0	14.5
	100	−5.5	105.7	13.9	13.3
	200	−0.4	102.7	11.8	14.7

RSD: relative standard deviation.
